# Using routinely collected data to enhance long term follow up data: an example from the building blocks trial

**DOI:** 10.1186/1745-6215-14-S1-P136

**Published:** 2013-11-29

**Authors:** Rebecca Cannings-John, Michael Robling

**Affiliations:** 1 South East Wales Trials Unit, Cardiff University, Cardiff, UK

## 

Non-responders or participants lost to follow-up can be problematic in trials especially those collecting long-term self-reported follow-up, resulting in missing data which incorporates bias in trial results. Linkage to routinely collected data can enhance the data held by RCTs. Building Blocks is a multi-centred individually RCT evaluating the effectiveness of the Family Nurse Partnership programme in 18 sites in England. The study will evaluate the effectiveness of FNP and usual care versus usual care for nulliparous pregnant women <20years, recruited by 24weeks gestation and followed until the child's second birthday. Self-reported data has been collected from participants at baseline, 34-36weeks gestation, 6, 12, 18 and 24months following birth.

To enhance self-reported data, routine data will be collected from a number of sources: maternity, abortions, primary and secondary care. This population of teenage mothers is a mobile and potentially vulnerable population and that increases the difficulty of follow-up. Therein lays the associated advantage of using routine data sources in that outcomes are verifiable and not just based on self-report.

This presentation will examine the use of data linkage for trial data to potentially enhance data quality, and to reduce costs over face-to-face data collection. It will document the processes of linking to different sources of routine data such as the governance issues surrounding gaining adequate consent for data linkage, linkage methods and quality control regarding linkage. We will also discuss optimising linkage in the way we collect trial data, and the potential for establishing broader linkage to other data sets.

